# The effects of season (spring versus autumn) on diagnosis of normocalcemic primary hyperparathyroidism

**DOI:** 10.3389/fendo.2022.1013362

**Published:** 2022-09-14

**Authors:** Magdalena Basińska-Lewandowska, Andrzej Lewiński, Krzysztof C. Lewandowski, Elżbieta Skowrońska-Jóźwiak

**Affiliations:** ^1^ ”Your Family Doctor”, General Practice Surgery, Lodz, Poland; ^2^ Department of Endocrinology and Metabolic Diseases, Medical University of Lodz, Lodz, Poland; ^3^ Department of Endocrinology and Metabolic Diseases, Polish Mother’s Memorial Hospital - Research Institute, Lodz, Poland

**Keywords:** normocalcemic primary hyperparathyroidism, vitamin D, screening, 25OHD, PTH

## Abstract

**Background:**

Raised parathormone (PTH) and normal calcium concentrations can be observed both in normocalcemic primary hyperparathyroidism (nPHPT) and in secondary hyperparathyroidism, e.g. due to vitamin D deficiency. We assessed the impact of season on the validity of diagnosis of nPHPT in terms of screening investigations to be performed in the primary care settings.

**Material and methods:**

On two occasions (March/April & September/October) we measured vitamin D (25OHD), PTH and total calcium in 125 healthy subjects, age range 6-50, not taking any vitamin D supplements.

**Results:**

In autumn there was an increase in 25OHD concentrations (from 18.1 ± 7.37ng/ml to 24.58 ± 7.72ng/ml, p<0.0001), a decline in PTH from 44.40 ± 17.76pg/ml to 36.63 ± 14.84pg/ml, p<0.001), without change in calcium levels. Only 45 subjects (36%) were vitamin D sufficient (25OHD>20/ml) in spring versus 83 (66.4%) in autumn, p<0.001. Elevated PTH concentrations were noted in 10 subjects in spring (8%) and in six subjects (4.8%) (p<0.05) in autumn. In spring, however, eight out of ten of these subjects (80%) had 25OHD<20 ng/ml, versus one in six (16.7%) in autumn (p<0.01). Normalization of PTH was observed in seven out ten subjects (70%), and all of them had 25-OHD<20 ng/ml in spring.

**Conclusions:**

In spring elevated PTH concentrations in the setting of normocalcemia are more likely to be caused by 25OHD deficiency rather by nPHPT. In contrast, in autumn, increased PTH concentrations are more likely to reflect nPHPT. We postulate that screening for nPHPT should be done in 25OHD replete subjects, i.e. in autumn rather than in spring.

## Introduction

Primary hyperparathyroidism (PHPT) is a well-defined disorder characterized by hypercalcemia and elevated levels of parathyroid hormone (PTH). It is one of the most common endocrine disorders with an estimated prevalence of 0.23% in women and 0.085% in men (1). Normocalcemic primary hyperparathyroidism (nPHPT) is one of newer presentation of primary hyperparathyroidism, in which PTH is elevated but serum calcium is normal. It is characterized by relatively high prevalence, varying between 0.1% up to 8,9% (2). Though, nPHPT is a recognized clinical entity (3), the optimal management still remains controversial (4, 5). Raised PTH concentrations may be also observed in the setting of low vitamin D (25OHD) levels, as well is in several other conditions, e.g. renal impairment, treatment with lithium salts, etc. The cut-points for 25OHD sufficiency are controversial and a 30 ng/ml (75 nmol/l) as suggested by the Endocrine Society is widely criticized, while a 20 ng/ml (50 nmol/l) sufficiency cut-off is suggested to be more appropriate (6). Classical PHPT is very commonly associated with 25OHD deficiency. For instance, Walker MD et al. (7) reported 25OHD deficiency (defined as concentrations <20 ng/ml) in 19% of patients with PHPT, but prevalence as high as 86% were observed in winter in a study from Denmark (average latitude 55-57 °N, i.e. only marginally higher than Poland - 50-54 °N), and 25OHD deficiency persisted in 77% subjects with PHPT also during summer (8). Furthermore, 25OHD deficiency was much more common in subjects with PHPT than in healthy controls, who also showed marked seasonal variations in concentrations of 25OHD (8). It was suggested that lower vitamin D binding protein levels is one of contributing mechanisms of low total 25(OH)D in PTHP patients (9). Wide seasonal variation of vitamin D concentrations is well documented in Polish population (10). Apart from variations in 25OHD, seasonal variation of PTH is also well recognized. For instance, in a large study from Romania (n=8409 subjects, average latitude 44-48°N) there was an average 9.36% fall in PTH concentrations (from 47.61 pg/ml to 43.15 pg/ml) from March to September (11). The study from Sweden (latitude 55-67° N) showed about 7% fall in PTH in summer (12), however without any significant change in calcium concentrations (change about 1%, i.e. not statistically significant). In a large study of 792 subjects with frank PHPT (serum calcium>10.5 mg%) there was about 8.4% decrease in PTH concentrations in summer in comparison to winter (13), though such variation seems to be attenuated by vitamin D supplementation (14). On the other hand, to the best of our knowledge, there are no data either on seasonal variations of PTH concentrations in nPHPT, or on the impact of such variability on the validity of the diagnosis of nPHPT. Currently testing of calcium, 25OHD and PTH concentrations is often performed in primary care, where differentiation of genuine nPHPT from cases related to 25OHD deficiency might be quite difficult (15). Furthermore, overdiagnosis of possible nPHPT in primary care settings would result in unnecessary specialist referrals, thus increasing the burden for state insurance institutions. Hence, in our study we have endeavored assess optimal timing for screening for possible nPHPT as a screening procedure to be performed in primary care settings. Indeed, the aim of the study was to select patients who would benefit from subsequent specialist referral.

## Subject and methods

The study included 125 subjects (56 males) age range 6-50 years, recruited from a single urban general practice – Your Family Doctor (single center study) in the city of Lodz (central Poland, latitude 51°75’ N). We have chosen this age range as these subjects usually do not take any 25OHD supplements. On the other hand, younger children and women above 50 (menopausal age) often take additional 25OHD, that would be a source of bias in keeping with the observations of Cong E, et al. (14).

As the study was performed in primary care settings then all these patients had their records dating back up to 20 years, and in younger patients since their birth. All subjects had no history of any kidney or liver disease, were not vegetarians, and were not taking any vitamin D supplements, thiazide diuretics, steroids, antiepileptic or osteoporotic medications. Pregnant or beast-feeding women were not included into the study. Our patients were also free of any major disabilities that could impede their stay outdoors. Demographic characteristics of subjects participating in the study is presented in **Table 1**.

**Table 1:** Demographic characteristics of subjects participating in the study.

Total number of participants	125
Males	56
Females	69
Age range	6-50
Age (mean ± SD) [years]	29.16 ± 13.24
BMI (mean ± SD) [kg/m^2^]	25.32 ± 6.33
BMI (mean ± SD) males [kg/m^2^]	25.06 ± 6.12
BMI(mean ± SD) females [kg/m^2^]	25.53 ± 6.52
Mean age males [years]	26.72 ± 13.36
Mean age females [years]	30.41 ± 14.58

Concentrations of 25OHD, parathormone (PTH) and total calcium were assessed twice in the same subjects in late winter/early spring (March-April), and late summer/early autumn (September-October). The reference range for total calcium was 8.41-10.42 mg/dl (2.1-2.6 mmol/l). We have chosen the measurements of total instead of ionized calcium as assessment of ionized calcium is a demanding procedure, sensitive to pH of blood (acidosis increase ionized calcium concentration) and preanalytical errors (fist-clenching or use of venous stasis may increase level of ionized calcium), requiring immediate handling and good quality control (16). This choice of screening tests was therefore, in our opinion, suitable for screening in primary care settings.

25OHD was measured by the means of Elecsys Vitamin D Total II assay, using Cobas 801 analyzer (Roche^®^) with intra-assay variation 1.1-3.1% and inter-assay variation 2.2-4.3%, while 1-84 amino acid PTH was measured by electrochemiluminescence ECLIA method - Elecsys system, Cobas 801 analyzer with intra-assay variation 1.7-2.6%, inter-assay variation 2.5-3.1%. The reference range for PTH, according to this assay, is 15-65 pg/ml. Increased PTH concentration was defined as a value above upper reference limit (65 pg/ml).

This study was conducted according to the guidelines laid down in the Declaration of Helsinki, and all procedures involving research study participants were approved by the Ethics Committee of the Polish Mother’s Memorial Hospital Research Institute, Lodz, Poland, decision nr 100/2019. Informed consent was obtained from all subjects involved in the study.

### Statistical analysis

The MedCalc 19.0.7 package was used for statistical analysis. Shapiro-Wilk and D’Agostino-Pearson tests were used to test the normality of distributions. The t-Student, Mann-Whitney and chi^2^ methods were used to compare parameters. The p<0.05 ratio was taken as statistically significant.

## Results

Changes in 25OHD, PTH and total calcium are presented in **Table 2**. There was an increase in 25OHD concentrations from spring to autumn from 18.1 ± 7.37 ng/ml (median 17.0 ng/ml) to 24.58 ± 7.72 ng/ml (median 23.1 ng/ml), p<0.0001, and a decline in PTH concentrations from 44.40 ± 17.76 pg/ml (median 41.65 pg/ml) to 36.63 ± 14.84 pg/ml (median 33.50 pg/ml), p<0.0001, without significant change in calcium concentrations 9.53 ± 0.41 mg/dl, *versus* 9.49 ± 0.76 mg/dl, p=0.65. All subjects had normal kidney function (mean creatinine 0.78 mg/dl ± 0.21 mg/dl) and total calcium concentrations within the reference range. There were no sex-related differences in BMI (25.06 ± 6.12 kg/m^2^ versus 25.53 ± 6.52 kg/m^2^) or age (26.72 ± 13.36 years versus 30.41 ± 14.58 years, for males and females, respectively).

**Table 2:** Concentrations of 25OHD, parathormone (PTH) and total Calcium (mean ± SD) in 125 healthy subjects not taking any 25OHD supplements tested twice in spring and in autumn from a single urban general practice in the city of Lodz, Poland, latitude 51°75’ N.

	March/April	September/October	P value
**25OHD [ng/ml]**	18.1 ± 7.37	24.58 ± 7.72	<0.0001
**PTH [pg/ml]**	44.40 ± 17.76	36.63 ± 14.84	<0.0001
**Total calcium [mg/dl]**	9.53 ± 0.41	9.49 ± 0.76	0.65

Only 45 subjects (36%) were vitamin D sufficient (i.e. 25OHD>20/ml) in March/April versus 83 (66.4%) in September/October, p<0.001. Elevated PTH concentrations were noted in 10 subjects in spring (8%), and in six subjects in autumn (4.8%, p<0.05).

Analysis of subjects with elevated PTH stratified according to 25OHD concentrations is presented in **Table 3**, while evolution of individual concentrations of PTH, 25OHD and calcium is presented in **Table 4**, in keeping with Institute of Medicine guidelines (17) 25OHD sufficiency was defined as concentrations above 20 ng/ml (50 nmol/L), with 25OHD deficiency being only present for concentrations below 12 ng/ml (30 nmol/L), while 25OHD insufficiency was defined for concentrations 12-20 ng/ml (30-50 nmol/L). Similar cut-off point was also used in other studies (7, 8).

**Table 3:** The number of patients with raised PTH concentrations (65 pg/ml) and normocalcemia depending on the season of the year and 25OHD status.

PTH > 65 pg/ml(whole population: n=125, age 6-50, reference range for PTH: 15-65 pg/ml)				
	Spring	Autumn	PTH normalization after summer	PTH increase above reference range after summer
TOTAL	10 (8.0%)	6 (4.8%)	7 (5.6%)	4 (3.2%)
25OHD >20 ng/ml	2 (1.6%)	5 (4.0%)	–	3 (2.4%)
25OHD 12-20 ng/ml	6 (4.8%)	1 (0.8%)	5 (4.0%)	1 (0.8)
25OHD <12 ng/ml	2 (1.6%)	0	2 (1.6%)	–

**Table 4:** Individual concentrations of PTH, 25OHD and total Calcium of patients with raised PTH (either in March/April, n=10, or in September/October, n=6), the fall in PTH (spring versus autumn) and an increase in 25OHD were statistically significant (p<0.001).

SPRING (March-April)					AUTUMN (September-October)					
**Nr**	**Age [years]** **Sex** **[F/M]**	**PTH** **[pg/ml]**	**25OHD** **[ng/ml]**	**Calcium** **[mg/dl]**	**PTH** **[pg/ml]**	**increment** **or decrement** **[%]**	**25OHD** **[ng/ml]**	**increment** **or decrement** **[%]**	**Total** **Calcium** **[mg/dl]**	**increment** **or decrement** **[%]**
1.	39F	**95.1**	30.0	9.33	44.2	**-53.5**	31.8	3.3	9.00	-3.5
2.	43F	**77.3**	8.1	9.30	57.9	**-25.1**	28.3	246	8.98	-3.4
3.	43F	**102.0**	12.1	8.58	60.1	**-41.1**	19.0	57	9.30	3.7
4.	40F	**66.7**	12.7	8.93	**74.1**	11.1	18.6	46	9.60	7.5
5.	32M	**79.8**	12.2	9.22	53.6	**-32.8**	33.0	170	9.50	3.0
6.	30F	**76.9**	25.0	9.20	**81.1**	5.5	28.2	13	9.10	1.1
7.	46F	**73.2**	16.1	9.11	37.2	**-39.6**	15.6	-3	9.15	0.4
8.	49F	**66.9**	8.1	9.05	45.7	**-31.6**	16.2	100	9.00	-0.5
9.	50M	**137.0**	13.0	9.25	**92.8**	**-32.3**	23.2	78	9.20	-0.5
10.	49F	**88.1**	15.2	9.20	**88.8**	0.8	23.3	53	9.40	2.2
11.	40F	61.5	9.0	9.90	**66.7**	8.4	25.6	184	9.91	0.1
12.	13F	64.6	15.6	9.68	**80.4**	24.4	24.1	54	9.80	1.2
**Mean** **± SD**		84.4 ± 21.2	14.7 ± 6.6	9.23 ± 0.33	65.2 ± 18.3	–	23.9 ± 5.8	–	9.32 ± 0.31	–

Raised PTH values as well PTH decrements after in September/October versus March/April are highlighted in bold.

Notably in the majority, i.e. in seven out of ten patients with high PTH concentrations in spring, we observed normalization of PTH concentrations in autumn. This phenomenon, however, pertained only to individuals with 25OHD insufficiency (12-20 ng/ml) or deficiency (25OHD<12 ng/ml), while in those with normal 25OHD concentrations (i.e. >20 ng/ml), there was indeed an increase in PTH concentrations in three out of five subjects, despite an overall decrease in PTH in the whole investigated cohort. Indeed, in eight out of ten subjects in spring (80%) elevated PTH concentrations were noted in the setting of 25OHD deficiency or insufficiency, while in autumn 25OHD insufficiency was noted only in one out of six subjects with high PTH (p<0.05, spring versus autumn). Hence, increased PTH concentrations in the setting of normocalcemia and 25OHD sufficiency were finally present in five subjects (4.0% of the investigated cohort).

## Discussion

Our study indicates that screening for normocalcemic primary hyperparathyroidism should be preferably done in autumn, as elevated PTH levels in spring are more likely to be related to 25OHD deficiency. Though our data are based on relatively small cohort (n=125), our results, are, however, quite convincing, given that in seven out 10 subjects in spring (i.e. 70%), increased PTH concentrations were indeed a transient phenomenon and reverted to normal in autumn. Given the average several month waiting time for specialist assessment, this would imply that seven out of ten patients might be unnecessarily referred to specialist clinic, thus generating additional costs as well as at least two unnecessary specialist visit for each patient (investigations are usually ordered during the first visit and reviewed on the second visit prior to possible discharge). Indeed, our study showed that patients are twice as likely to be 25OHD sufficient in September-October than in March/April (66.4% versus 36%). Though, to best of our knowledge, there are no data on seasonal variation of PTH in nPHPT, in our opinion, our observations are corroborated by results of the study by Das G et al. (18) that was performed in a specialist clinic settings in Wales (UK) – latitude 53-54°N, similar to northern Poland. The Authors investigated patients with elevated PTH and 25OHD deficiency, who were referred to their clinic. They eventually diagnosed nPHPT in 23 subjects out of 111 (20.7%) and frank PHPT in 16 subjects (14.4%) with 25OHD concentrations below 20 ng/ml. That means that in 64.9% of patients (72 out of 111) elevated PTH was most likely caused by 25OHD deficiency, rather than by frank or normocalcemic PHPT.

Our results are also in keeping with observations of Shini et al. (19) who analyzed data obtained from 6280 patients from Sheffield area (UK) between 2013 and 2018. Indeed, the authors suggest that increased PTH concentrations in the setting of normocalcemia, normal kidney function and 25OHD above 20 ng/ml are more likely to indicate primary hyperparathyroidism, while secondary hyperparathyroidism should be suspected for 25OHD concentrations <20 ng/ml.

Recently, the significance of commercial assay type on the diagnosis of nPHPT has been shown (20). Namely, Abbott iPTH results were 53.4% (IQR 42.3–67.1, p<0.001) and 21.4% (IQR 12.1–28.1, p<0.001) higher than Roche iPTH and Siemens iPTH results, respectively. Siemens iPTH results were 26.8% (IQR 19.6–37.9, p<0.001) higher than Roche iPTH results. Hence, the use of Roche assay in our study might result in underdiagnosis rather than overdiagnosis of nPHPT. Moreover, PTH reference ranges are not adjusted for age (16), what is important since higher PTH levels were observed in elderly people (21), who were, however, not included in our study.

Furthermore, our data point to rather high prevalence of possible nPHPT in urban Polish population (around 4.0-4.8% in autumn). Prevalence on nPHPT is reported within a very wide range, depending on the selection criteria (2), though timing of the screening in relation to season of the year was not mentioned. Important factor affecting nPHPT diagnosis is a definition of 25OHD sufficiency, which is debatable (6). For instance, Vignali et al. (22) reported the prevalence of nPHPT in seven out of 685 (1.02%) of fully evaluated subjects from a village in southern Italy, but with 25OHD sufficiency cut-off of 30 ng/ml, and without specification of the timing of sample collection. Though discussion whether 20 ng/ml or 30 ng/ml 25OHD sufficiency cut-off is most optimal was beyond the scope of our study, it should be mentioned that relationship between 25OHD concentrations and PTH becomes relatively flat for 25OHD concentrations above 20 ng/ml. Namely, Malabanan et al. (23) demonstrated that an expected decrease in PTH, when serum vitamin D concentration was increased by administration of 50 000 IU of 25OHD once weekly for 8 weeks, was present only in subjects with a baseline 25OHD level of less than 20 ng/ml. Moreover, Sai et al. (24) suggest that vitamin D insufficiency should be defined as serum 25OHD less than 20 ng/mL as it relates to the bone.

The main limitation of our study is related to the lack of assessment of 24-hour urinary calcium and creatinine excretion and calcium-to-creatinine clearance ratio. Indeed, some of our subjects with raised PTH concentrations in autumn might have a variant of Familial Hypocalciuric Hypercalcemia (i.e. raised PTH with total calcium at the upper reference range). To the best of our knowledge there are no data on prevalence of FHH in Polish population. Also, the measurements of phosphorus might be useful in a differential diagnosis between nPHPT and secondary hyperparathyroidism due to 25OHD deficiency as suggested by Guo Y et al. (15). The diagnosis of nPHPT is established after exclusion of secondary causes of hyperparathyroidism, such as renal disease, vitamin D deficiency, malabsorption and dietary calcium deficiency or use of medications that may increase PTH level (i.e. bisphosphonates, denosumab, loop diuretics, antiepileptic drugs, lithium) (16), and virtually all of these conditions were excluded in our patients. Other rare causes of elevated PTH like pseudohypoparathyroidism should also be excluded, but none of our patients presented with very high PTH in the setting of hypocalcemia. Though diagnostic algorithm applied in our study could have certainly been improved, it reflects the real life situation in primary care settings. In that sense, our study was not designed to establish the final diagnosis, but to identify patients who would benefit from specialist referral and further detailed investigations. Indeed, subjects with raised PTH and suspected nPHPT can be referred for further specialist assessment in order to either confirm or refute the diagnosis. Yet, in our opinion, many of such referrals might be superfluous, if their PTH concentrations normalize in summer, following an expected increase in 25OHD concentrations, i.e. by the time when then reach the specialist clinic.

In summary, our study shows that the likelihood to obtain the correct diagnosis of possible nPHPT is amplified if patients are evaluated after correction of 25OHD deficiency. In subjects not taking vitamin D supplements this is much more likely to be achieved in autumn, when patients are twice as likely to be vitamin D sufficient. Further research is needed either to confirm, or to refute quite high prevalence (4%) of possible nPHPT in young and middle-age Poles in urban settings.

## Data availability statement

The raw data supporting the conclusions of this article will be made available by the authors, without undue reservation.

## Ethics statement

The studies involving human participants were reviewed and approved by Ethics Committee of the Polish Mother’s Memorial Hospital - Research Institute, Lodz, Poland (approval code no.: 100/2019). Written informed consent to participate in this study was provided by all participants or their legal guardian/next of kin.

## Author contributions

MB-L conceived the idea of the present study, collected of data at “Your Family Doctor”, General Practice Surgery, Lodz, Poland and prepared the manuscript; AL mentored writing of the manuscript; KL analyzed data; ES-J provided advice on the analysis process and supervised the final version of the manuscript. All authors contributed to the article and approved the submitted version.

## Funding

The study was supported by statutory funds from the Medical University of Lodz (503/1–107-03/503–11-001–19-00) and the Polish Mother’s Memorial Hospital - Research Institute, Lodz, Poland.

## Conflict of interest

The authors declare that the research was conducted in the absence of any commercial or financial relationships that could be construed as a potential conflict of interest.

## Publisher’s note

All claims expressed in this article are solely those of the authors and do not necessarily represent those of their affiliated organizations, or those of the publisher, the editors and the reviewers. Any product that may be evaluated in this article, or claim that may be made by its manufacturer, is not guaranteed or endorsed by the publisher.

## References

[1] Yeh MW Ituarte PH Zhou HC Nishimoto S Liu IL Harari A. Incidence and prevalence of primary hyperparathyroidism in a racially mixed population. J Clin Endocrinol Metab (2013) 98(3):1122–9. doi: 10.1210/jc.2012-4022 PMC3590475 23418315

[2] Pawłowska M Cusano NE. An overview of normocalcemic primary hyperparathyroidism. Curr Opin Endocrinol Diabetes Obes (2015) 22(6):413–21. doi: 10.1097/MED.0000000000000198 26512768

[3] Eastell R Arnold A Brandi ML D'Amour P Hanley DA Sudhaker Rao D. Diagnosis of asymptomatic primary hyperparathyroidism: Proceedings of the third international workshop. J Clin Endocrinol Metab (2009) 94(2):340–50. doi: 10.1210/jc.2008-1758 19193909

[4] Salcuni AS Battista C Pugliesie F Columbu C Guarnieri V Carnevale V. Normocalcemic primary hyperparathyroidism: an update. Minerva Endocrinol (Torino) (2021) 46(3):262–71. doi: 10.23736/S0391-1977.20.03215-0 33103871

[5] Aojula N Khan S Gittoes N Hassan-Smith Z. Normocalcaemic primary hyperparathyroidism: What is the role of parathyroid surgery? Therap Adv Endocrinol Metab (2021) 12:1–10. doi: 10.1177/2042018821995370 PMC7923978 33717430

[6] Bouillon R Van Schoor NM Gielen E Boonen S Mathieu C Vanderschueren D. Optimal vitamin D status: A critical analysis on the basis of evidence-based medicine. J Clin Endocrinol Metab (2013) 98(8):E1283–304. doi: 10.1210/jc.2013-1195 23922354

[7] Walker MD Cong E Lee JA Kepley A Zhang C McMahon DJ. Vitamin D in primary hyperparathyroidism: Effects of clinical, biochemical and densitometric presentation. J Clin Endocrinol Metab (2015) 100(9):3443–51. doi: 10.1210/jc.2015-2022 PMC4570160 26079779

[8] Moosgard B Vestergaard P Heikendorff L Melsen F Christiansen P Mosekilde L. Vitamin D status, seasonal variations, parathyroid adenoma weight and bone mineral density in primary hyperparathyroidism. Clin Endocrinol (Oxf) (2009) 5(63):506–13. doi: 10.1111/j.1365-2265.2005.02371x 16268801

[9] Wang X Sheng Z Meng L Su C Trooskin S Shapses SA. 25-hydroxyvitamin d and vitamin D binding protein levels in patients with primary hyperparathyroidism before and after parathyroidectomy. Front Endocrinol (Lausanne) (2019) 27:171. doi: 10.3389/fendo.2019.00171 PMC6446311 30972023

[10] Chlebna-Sokół D Konstantynowicz J Abramowicz P Kulik-Rechberger B Niedziela M Obuchowicz A. Evidence of a significant vitamin D deficiency among 9-13-year-old polish children: Results of a multicentre study. Eur J Nutr (2019) 58(5):2029–36. doi: 10.1007/s00394-018-1756-4 PMC6647701 29936536

[11] Niculescu DA Deacu LG Caragheorgeopol A Dusceac R Procopic C Petris R. Seasonal periodicity of serum parathyroid hormone and its relation to vitamin D in Romania. Arch Osteoporos (2020) 15(1):66. doi: 10.1007/s11657-020-00744-1 32367244

[12] Piirainen R Englund E Henriksson AE. The impact of seasonal variations of 25-hydroxyvitamin D and parathyroid hormone on calcium levels. Clin Biochem (2016) 49(12):850–3. doi: 10.1016/j.clinbiochem.2016.06.009 27343767

[13] Nevo-Shor A Kogan S Joshua BZ Bahat-Dinur A Novack V Fraenkel M. Seasonal changes in serum calcium, PTH and vitamin D levels in patients with primary hyperparathyroidism. Bone (2016) 89:59–63. doi: 10.1016/j.bone.2016.05.012 27260647

[14] Cong E Walker MD Kepley A Zhang C McMahon DJ Silverberg SJ. Seasonal variation in vitamin D levels no longer detectable in primary hyperparathyroidism. J Clin Endocrinol Metab (2015) 100(9):3452–9. doi: 10.1210/JC.2015-2105 PMC4570170 26120793

[15] Guo Y Wang Q Lu C Fan P Li J Luo X. New parathyroid function index for the differentiations of primary and secondary hyperparathyroidism: a case control study. Endocr Disord (2020) 20(1):5. doi: 10.1186/s12902-019-0487-8 PMC6950802 31914999

[16] Muñoz de Nova JL Sampedro-Nuñez M Huguet-Moreno I Marazuela Azpiroz M. A practical approach to normocalcemic primary hyperparathyroidism. Endocrine (2021) 74(2):235–44. doi: 10.1007/s12020-021-02845-4 34386939

[17] Rosen CJ Abrams SA Aloia JF Brannon PM Clinton SK Durazo-Arvizu RA. IOM committee members respond to endocrine society vitamin D guideline. J Clin Endocrinol Metab (2012) 97:1146–52. doi: 10.1210/jc.2011-2218 PMC5393439 22442278

[18] Das G Eligar V Govindan J Bondugulapati LNR Okosienne O Davies S. Impact of vitamin d replacement in patients with normocalcaemic and hypercalcaemic primary hyperparathyroidism and coexisting vitamin D deficiency. Ann Clin Biochem (2015) 52(Pt4):462–9. doi: 10.1177/0004563214564400 25468998

[19] Schini M Jacques RM Oakes E Peel NFA Walsh JS Eastell R. Normokalcemic hyperparathyroidism: Study of its prevalence and natural history. J Clin Endocrinol Metab (2020) 105:e1171–86. doi: 10.1210/clinem/dgaa084 PMC7069345 32072184

[20] Kalaria T Fenn J Sanders A Yates A Duff C Ashby H. The diagnosis of normocalcaemic hyperparathyroidism is strikingly dissimilar using different commercial laboratory assays. Horm Metab Res (2022) 54(7):429–34. doi: 10.1055/a-1856-4900 35835142

[21] Delgado JA Bauça JM Pastor MI Barceló A. Use of data mining in the establishment of age-adjusted reference intervals for parathyroid hormone. Clin Chim Acta (2020) 508:217–20. doi: 10.1016/j.cca.2020.05.030 32417213

[22] Vignali E Cetani F Chiavistelli S Meola A Saponaro F Centoni R. Normocalcemic primary hyperthyroidism: a survey in a small village of southern Italy. Endocr Connect (2015) 4(3):172–8. doi: 10.1530/EC-15-0030 PMC4496527 26155986

[23] Malabanan A Veronikis IE Holick MF. Redefining vitamin D insufficiency. Lancet (1998) 351(9105):805–6. doi: 10.1016/s0140-6736(05)78933-9 9519960

[24] Sai AJ Walters RW Fang X Gallagher JC. Relationship between vitamin D, parathyroid hormone, and bone health. J Clin Endocrinol Metab (2011) 96(3):E436–46. doi: 10.1210/jc.2010-1886 PMC3047227 21159838

